# A Tail Does Not Always Make a Difference: Assembly of *cds* Nets from Co(NCS)_2_ and 1,4-bis(*n*-Alkyloxy)-2,5-bis(3,2′:6′,3″-terpyridin-4′-yl)benzene Ligands

**DOI:** 10.3390/molecules27154995

**Published:** 2022-08-05

**Authors:** Simona S. Capomolla, Giacomo Manfroni, Alessandro Prescimone, Edwin C. Constable, Catherine E. Housecroft

**Affiliations:** Department of Chemistry, University of Basel, BPR 1096, Mattenstrasse 24a, CH-4058 Basel, Switzerland

**Keywords:** 3,2′:6′,3″-terpyridine, cobalt(II) thiocyanate, coordination network, solvent effects

## Abstract

The consistent assembly of a (6^5^.8) *cds* net is observed in reactions of cobalt(II) thiocyanate with 1,4-bis(*n*-alkyloxy)-2,5-bis(3,2′:6′,3″-terpyridin-4′-yl)benzene ligands in which the *n-*alkyloxy substituents are *n*-propyl (ligand **3**), *n*-butyl (**4**), *n*-pentyl (**5**), *n*-hexyl (**6**), *n*-heptyl (**7**), and *n*-octyl (**8**). Crystals were grown by layering a methanol solution of Co(NCS)_2_ over a 1,2-dichlorobenzene solution of each ligand. The choice of crystallization solvents is critical in directing the assembly of the *cds* net. Single-crystal structures of [Co(NCS)_2_(**3**)]*_n_***^.^**3.5*n*C_6_H_4_Cl_2_, [Co(NCS)_2_(**4**)]_n_**^.^**5.5*n*C_6_H_4_Cl_2_, [Co(NCS)_2_(**5**)]*_n_***^.^**4*n*C_6_H_4_Cl_2_, [Co(NCS)_2_(**6**)]*_n_***^.^**3.8*n*C_6_H_4_Cl_2_, [Co(NCS)_2_(**7**)]*_n_***^.^**3.1*n*C_6_H_4_Cl_2_, and [Co(NCS)_2_(**8**)]*_n_***^.^**1.6*n*C_6_H_4_Cl_2_**^.^**2*n*MeOH (C_6_H_4_Cl_2_ = 1,2-dichlorobenzene) are presented and compared. The *n-*alkyloxy chains exhibit close to extended conformations and are accommodated in cavities in the lattice without perturbation of the coordination framework.

## 1. Introduction

**Scheme 1:**
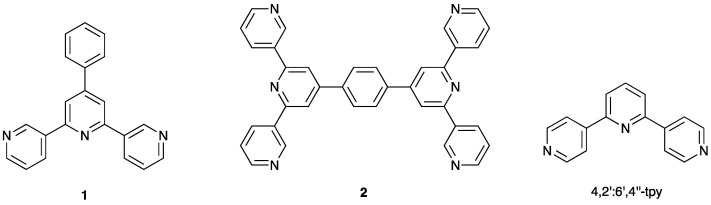
The structures of the ditopic ligand **1**, the first tetratopic ligand **2**, containing two 3,2′:6′,3″-tpy metal-binding domains, and 4,2′:6′,4″-terpyridine.

**Scheme 2:**
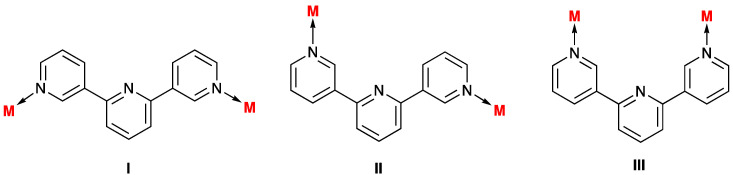
Limiting planar conformations of the 3,2′:6′:3″-tpy metal-binding domain. Only the outer pyridine rings coordinate to metal ions.

**Scheme 3:**
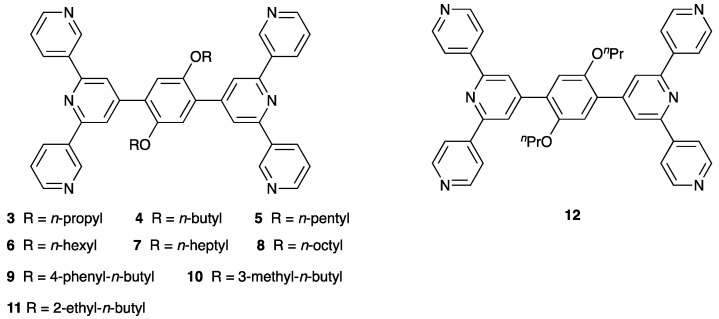
The structures of ligands **3**–**12**.

The first metal coordination compound containing the 3,2′:6′,3″-terpyridine (3,2′:6′,3″-tpy) domain was reported in 2008 by Grafino et al. and demonstrated metal binding by the outer pyridine rings of 4′-phenyl-3,2′:6′,3″-terpyridine (**1**, Scheme 1) in the discrete molecular complex [Zn_2_(μ-**1**)(acac)_4_]·H_2_O (Hacac = pentane-2,4-dione) [1]. This ditopic coordination environment is typical of 3,2′:6′,3″-tpy, although coordination through a single pyridine ring has also been observed, often when a coordinatively non-innocent functionality such as a carboxylate has been introduced into the 3,2′:6′,3″-tpy unit [2,3,4,5,6,7,8,9,10,11] or when protonation of a pyridine ring occurs [12,13]. Compared to 4,2′:6′,4”-tpy (Scheme 1), 3,2′:6′,3″-tpy is conformational flexible (Scheme 2), allowing the metal-binding domain to adapt to different environments imposed either by the coordination geometry of a metal center or to spatial constraints within the crystal lattice. However, it is important to note that changes in the conformation of the 3,2′:6′,3″-tpy units do not necessarily result in modification of the coordination network type [14].

Yoshida et al. [15] were the first to report the coordination chemistry of tetratopic ligands combining two divergent terpyridine domains; this included ligand **2** (Scheme 1). In addition to the conformational flexibility described in Scheme 2, ligands such as **2** also exhibit conformational variation arising from bond rotation about the arene spacer–tpy C–C bonds. With reference to the centroid of the central arene ring in **2** and the four outer pyridine N-donors, the ligand is described as a 4-connecting node, and the limiting geometries are defined with the two 3,2′:6′,3″-tpy units being coplanar or orthogonal [16].

In our recent investigations, we have been focusing on coordination networks assembled using combinations of [Cu(hfacac)_2_] (Hhfacac = hexafluoropentane-2,4-dione) and ligands related to **2** which contain *n*-alkyloxy substituents bonded to the central arene spacer [17]. The *n*-alkyloxy groups increase the solubility of the bis(3,2′:6′,3″-tpy) ligands, as well as providing a means of tuning the steric requirements of the ligands. Using ligands **3–8** (Scheme 3) as well as their methoxy and ethoxy-substituted analogs, we demonstrated the dominant assembly of (4,4) nets directed by the bis(3,2′:6′,3″-tpy) ligands acting as 4-connecting nodes. We showed that changes in conformation of the 3,2′:6′,3″-tpy units from **I** to **II** (Scheme 2) did not influence the network topology. Related to these observations is the reaction of ligand **2** with [Co(CNacac)_2_] (HCNacac = 3-cyanopentane-2,4-dione), which also leads to a (4,4)-net with the ligand functioning as a 4-connecting node and the metal center only acting as a linker [15].

By combining bis(3,2′:6′,3″-tpy) ligands with Co(NCS)_2_, both ligand and metal center have the potential to act as 4-connecting nodes. Typically, coordination of pyridine-based ligands with Co(NCS)_2_ yields *trans*-{Co(NCS)_2_(N)_4_} domains, which can function as a 4-connecting node in a coordination network. The coordination chemistry of bis(3,2′:6′,3″-tpy) ligands with Co(NCS)_2_ is still little explored and only four structures appear in the Cambridge Structural Database (CSD) version 2022.1.0 [18], searched using Conquest version 2022.1.0 [19]. However, even with these limited examples, three different networks are exemplified illustrating the flexibility of the bis(3,2′:6′,3″-tpy) building block. In each of the following, crystals were obtained under ambient conditions, using crystal growth by layering. A combination of a 1,2-Cl_2_C_6_H_4_ solution of **9** (Scheme 3) and a MeOH solution of Co(NCS)_2_ yielded [Co(NCS)_2_(**9**)]*_n_***^.^**1.6*n*H_2_O**^.^**1.2*n*C_6_H_4_Cl_2_, which assembled into a 3D (6^5^.8) *cds* net [20]. In contrast, use of CHCl_3_ solutions of **10** and **11** (Scheme 3) and a MeOH solution of Co(NCS)_2_ produced trinodal self-interpenetrating (6^2^.8^4^)(6^4^.8^2^)(6^5^.8)_2_ nets, with powder X-ray diffraction data confirming that the single crystal structures were representative of the bulk materials [21]. In each of these structures (CSD refcodes KOXJEP, NORVAU and NORVOI), the asymmetric unit contains either one or two, crystallographically independent half-ligands with the second half generated by inversion. Thus, each ligand in [Co(NCS)_2_(**9**)]*_n_***^.^**1.6*n*H_2_O**^.^**1.2*n*C_6_H_4_Cl_2_ [20], [Co(NCS)_2_(**10**)]*_n_***^.^***n*MeOH**^.^**3*n*CHCl_3_ [21] and [Co(NCS)_2_(**11**)]*_n_***^.^**0.8*n*MeOH**^.^**1.8*n*CHCl_3_ [21] is a planar, 4-connecting node, and in each structure, the 3,2′:6′,3″-tpy domain adopts conformation **II** (Scheme 2). We have also observed that layering of a MeOH solution of Co(NCS)_2_ over a CHCl_3_ solution of **8** (Scheme 3) resulted in the assembly of a 3D (4^2^.8^4^)(4^2^.8^4^) net (CSD refcode LOTDIJ) [22] directed by a combination of planar, 4-connecting Co nodes and pseudo-tetrahedral ligand nodes (i.e., ligand **8** adopts a conformation in which the two 3,2′:6′,3″-tpy units approach orthogonality). In [Co(NCS)_2_(**8**)]*_n_***^.^**4*n*CHCl_3_, ligand **8** adopts conformation **I** (Scheme 2). These latter results motivated us to further investigate network assembly using Co(NCS)_2_ and bis(3,2′:6′,3″-tpy) ligands, and we chose to use ligands **3**–**8** with a common solvent combination of methanol and 1,2-dichlorobenzene. A reason for investigating the series **3**–**8** is that we have previously observed changes in coordination assembly as a result of varying the length of the *n*-alkyloxy chain in series of 3,2′:6′,3″-tpy and 4,2′:6′,4″-tpy ligands [23,24,25,26].

## 2. Results and Discussion

### 2.1. Crystal Growth Conditions

We have previously reported the synthesis and characterization of compounds **3**–**8**. Crystal growth experiments were carried out under ambient conditions (ca. 22 °C) by layering a MeOH solution of Co(NCS)_2_ over a 1,2-Cl_2_C_6_H_4_ solution of each ligand. Reactions were carried out on the same scale with the same concentrations of solutions, and X-ray quality crystals were obtained in periods ranging from 21 days to two months (see Section 3).

### 2.2. Single Crystal Structures

**Figure 1:**
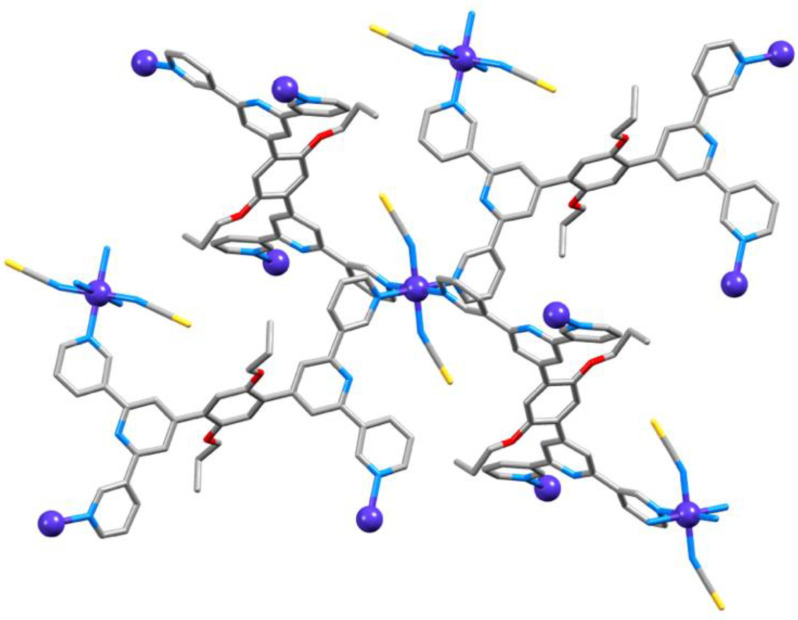
In [Co(NCS)_2_(**3**)]*_n_***^.^**3.5*n*C_6_H_4_Cl_2_, both the Co atom and ligand **3** act as 4-connecting nodes. H atoms and solvent molecules are omitted for clarity.

The compounds [Co(NCS)_2_(**3**)]*_n_***^.^**3.5*n*C_6_H_4_Cl_2_ and [Co(NCS)_2_(**4**)]_n_**^.^**5.5*n*C_6_H_4_Cl_2_ crystallized in the monoclinic space group *P*2_1_/*c*, while [Co(NCS)_2_(**5**)]*_n_***^.^**4*n*C_6_H_4_Cl_2_, [Co(NCS)_2_(**6**)]*_n_***^.^**3.8*n*C_6_H_4_Cl_2_, [Co(NCS)_2_(**7**)]*_n_***^.^**3.1*n*C_6_H_4_Cl_2_ and [Co(NCS)_2_(**8**)]*_n_***^.^**1.6*n*C_6_H_4_Cl_2_**^.^**2*n*MeOH crystallized in the monoclinic space group *P*2_1_/*n*. All six compounds exhibit similar, extended structures and we therefore discuss them together. The structures of the asymmetric units with atom numbering are shown in Figures S1–S6 in the Supporting Material. Each bis(3,2′:6′,3″-tpy) ligand binds to four different {Co(NCS)_2_} units and therefore functions as a 4-connecting node. Each Co(II) center is 6-coordinate and is in a *trans*-{Co(NCS)_2_(N)_4_} environment, coordinated by four different bis(3,2′:6′,3″-tpy) ligands. Thus, each Co(II) is also a 4-connecting node. Figure 1 illustrates this for [Co(NCS)_2_(**3**)]*_n_***^.^**3.5*n*C_6_H_4_Cl_2_ as representative of the six structures. Selected bond parameters are given in Table 1; the Co–N bond lengths are unexceptional, and the N –Co–N bond angles are all close to 90°, consistent with a regular octahedral coordination sphere.

**Figure 2:**
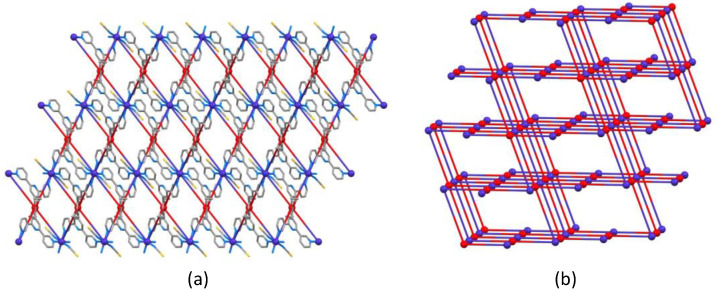
(**a**) The *cds* network in [Co(NCS)_2_(**3**)]*_n_***^.^**3.5*n*C_6_H_4_Cl_2_ viewed down the crystallographic *b*-axis. (**b**) The same network showing only the 4-connecting nodes.

The asymmetric unit in each of the structures of the complexes containing **3**, **5**, **6**, **7** and **8** contains half of one independent ligand, with the second half being generated by inversion (Figures S1 and S3–S6 in the Supporting Material). Thus, symmetry dictates that the four N-donors are coplanar. In [Co(NCS)_2_(**4**)]*_n_***^.^**5.5*n*C_6_H_4_Cl_2_, the asymmetric unit contains one whole and one-half independent ligands. For the latter, the ligand is completed by inversion and so, again, the four N-donors (N7, N9, N7^i^, N9^i^ in Figure S2) are coplanar. For the crystallographically independent ligand containing N1, N2, N3 and N4 (Figure S2), the angle between the plane containing N1, N2 and the centroid of the arene spacer, and the plane containing N3, N4 and the centroid of the arene ring, is 3.4°. This ligand is therefore also a planar, 4-connecting node, and while the ligands in [Co(NCS)_2_(**4**)]*_n_***^.^**5.5*n*C_6_H_4_Cl_2_ are crystallographically independent, they are topologically equivalent. Therefore, in all six structures, both the bis(3,2′:6′,3″-tpy) ligand and the cobalt center are planar, 4-connecting nodes and the assemblies propagate into 3D networks with a *cds* topology. This is one of the more common networks comprising planar 4-connecting nodes [27,28]; half of the adjacent nodes are coplanar and half are mutually perpendicular. This is shown for [Co(NCS)_2_(**3**)]*_n_***^.^**3.5*n*C_6_H_4_Cl_2_ in Figure 2, in which the ligand and metal nodes are shown in red and blue, respectively. The coordination networks with **3**–**8** are structurally related to that in [Co(NCS)_2_(**9**)]*_n_***^.^**1.6*n*H_2_O**^.^**1.2*n*C_6_H_4_Cl_2_, crystals of which were also grown using a MeOH/1,2-Cl_2_C_6_H_4_ mixture [20]. The *cds* net was also found for [Co(NCS)_2_(**12**)]*_n_***^.^**2*n*C_6_H_4_Cl_2_ (see Scheme 3 for ligand **12**) in which **12** is an isomer of **3** [29].

**Figure 3:**
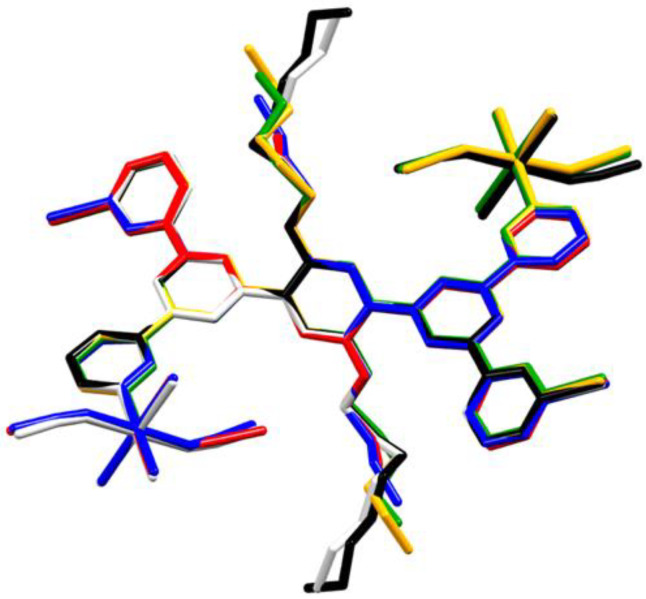
Overlay of the ligand conformations showing the four coordinated *trans*-{Co(NCS)_2_(N)_4_} units in [Co(NCS)_2_(**3**)]*_n_***^.^**3.5*n*C_6_H_4_Cl_2_ (red), [Co(NCS)_2_(**4**)]*_n_***^.^**5.5*n*C_6_H_4_Cl_2_ (blue), [Co(NCS)_2_(**5**)]*_n_***^.^**4*n*C_6_H_4_Cl_2_ (green), [Co(NCS)_2_(**6**)]*_n_***^.^**3.8*n*C_6_H_4_Cl_2_ (yellow), [Co(NCS)_2_(**7**)]*_n_***^.^**3.1*n*C_6_H_4_Cl_2_ (pale grey), and [Co(NCS)_2_(**8**)]*_n_***^.^**1.6*n*C_6_H_4_Cl_2_**^.^**2*n*MeOH (black). In [Co(NCS)_2_(**4**)]*_n_***^.^**5.5*n*C_6_H_4_Cl_2_, one of the two independent ligands is shown; both ligands have similar conformations (Table 2).

**Figure 4:**
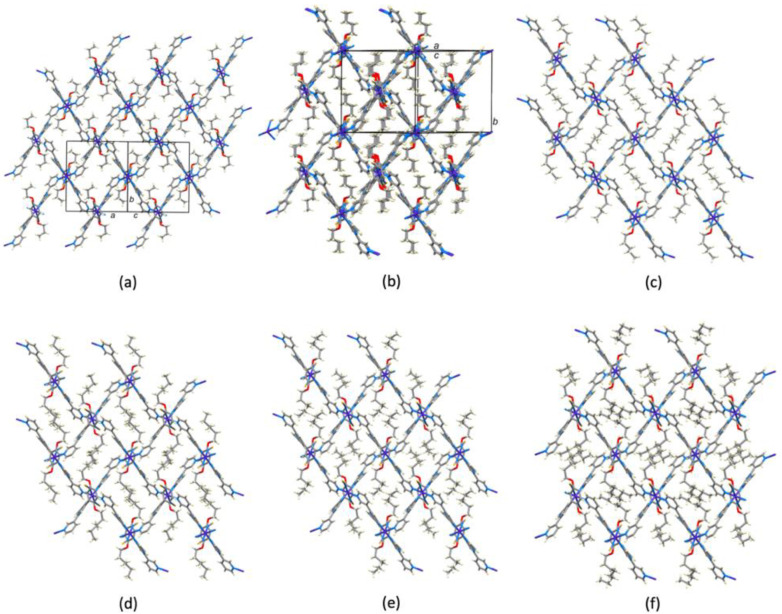
Packing diagrams of (**a**) [Co(NCS)_2_(**3**)]*_n_***^.^**3.5*n*C_6_H_4_Cl_2_, (**b**) [Co(NCS)_2_(**4**)]_n_**^.^**5.5*n*C_6_H_4_Cl_2_, (**c**) [Co(NCS)_2_(**5**)]*_n_***^.^**4*n*C_6_H_4_Cl_2_, (**d**) [Co(NCS)_2_(**6**)]*_n_***^.^**3.8*n*C_6_H_4_Cl_2_, (**e**) [Co(NCS)_2_(**7**)]*_n_***^.^**3.1*n*C_6_H_4_Cl_2_, and (**f**) [Co(NCS)_2_(**8**)]*_n_***^.^**1.6*n*C_6_H_4_Cl_2_**^.^**2*n*MeOH. Solvent molecules have been removed. In (**c**–**f**), the packing diagram is viewed down the *c*-axis. In (**b**), there are two crystallographically independent ligands.

In each compound, the 3,2′:6′,3″-tpy unit adopts conformation **II** (Scheme 2), and the angles between the least-squares planes of bonded pairs of aromatic rings in the coordinated ligands **3**–**8** show striking similarities (Table 2). This is further demonstrated in the overlay of one building block from each structure displayed in Figure 3. In each structure, the *n*-alkyloxy chains are in close to extended conformations and it is noteworthy that the increase in steric demands of the substituents has negligible effect on the overall structure as discussed below. Packing diagrams of the six structures with solvent molecules omitted are displayed in comparable orientations in Figure 4 and illustrate how the *n*-alkyloxy chains are accommodated in analogous cavities with little impact on the 3D framework. The solvent-accessible void space was calculated using Mercury version 2022.1.0 [30] with a contact surface map with probe radius of 1.2 Å. The decrease from 49.3% to 34.4% (Table 3) is consistent with the increase in the steric demands of the *n*-alkyloxy chains.

### 2.3. Bulk Sample Analysis

Powder X-ray diffraction (PXRD) and solid-state IR spectroscopy were used to analyze the bulk materials after single crystals had been selected for single-crystal X-ray diffraction. The IR spectra are shown in Figures S7–S12, and a strong absorption at 2065, 2069, 2069, 2067, 2068 or 2056 cm^−1^ for the compound containing ligands **3**, **4**, **5**, **6**, **7** or **8**, respectively, was assigned to the ν(CN) mode of the coordinated thiocyanato ligands. The fingerprint regions of the IR spectra are all similar.

**Figure 5:**
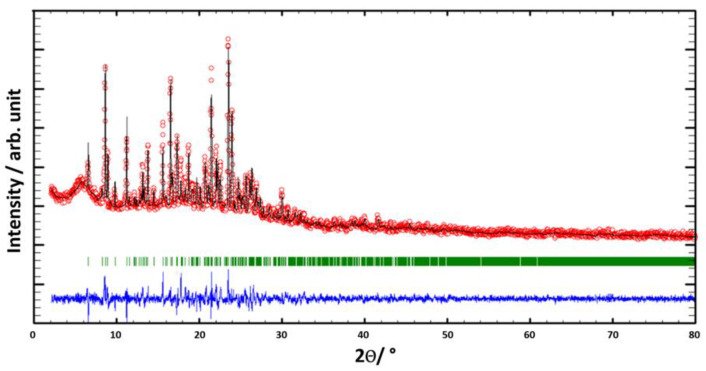
PXRD (CuKα1 radiation) patterns for [Co(NCS)_2_(**6**)]*_n_***^.^**3.8*n*C_6_H_4_Cl_2_. Experimentally obtained pattern (red circles) is compared with the best fit from the Rietveld refinement analysis (black line). Bragg peak positions (green) and differences between the calculated and experimental plots (blue) are also shown.

For [Co(NCS)_2_(**6**)]*_n_***^.^**3.8*n*C_6_H_4_Cl_2_, an excellent fit was found between the experimental PXRD pattern for the bulk material and the pattern predicted from the single-crystal structure (Figure 5). However, for the remaining compounds, good fits were not obtained, most likely because of solvent loss which occurs on standing at ambient temperatures. Overlays of the experimental PXRD (298 K) for the bulk material and that predicted from the single crystal structures (150 K) of [Co(NCS)_2_(**3**)]_n_**^.^**3.5nC_6_H_4_Cl_2_ and [Co(NCS)_2_(**4**)]_n_**^.^**5.5nC_6_H_4_Cl_2_ are shown in Figures S13 and S14.

For [Co(NCS)_2_(**3**)]_n_**^.^**3.5nC_6_H_4_Cl_2_, [Co(NCS)_2_(**4**)]_n_**^.^**5.5nC_6_H_4_Cl_2_ and [Co(NCS)_2_(**6**)]*_n_***^.^**3.8*n*C_6_H_4_Cl_2_, thermogravimetric analysis (TGA) coupled with mass spectrometry was used to investigate loss of solvent from the lattice upon heating. The results are summarized in Table 4. The crystals of the coordination polymers were heated to 210 °C. Loss of 1,2-dichlorobenzene was detected with mass peaks at *m*/*z* 146, 148 and 111 (arising from C_6_H_4_^35^Cl_2_^+^, C_6_H_4_^35^Cl^37^Cl^+^ and C_6_H_4_^35^Cl^+^). Detection at *m*/*z* 31.0 was used to check for loss of MeOH; none or a negligible amount was observed (Figures S15–S17). [Co(NCS)_2_(**4**)]*_n_***^.^**5.5*n*C_6_H_4_Cl_2_ and [Co(NCS)_2_(**6**)]*_n_***^.^**3.8*n*C_6_H_4_Cl_2_ exhibit loss of C_6_H_4_Cl_2_ in two steps, while [Co(NCS)_2_(**3**)]*_n_***^.^**3.5*n*C_6_H_4_Cl_2_ shows a three-step process (Figures S15–S17).

### 2.4. Role of Solvents

The consistency of the *cds* net in the [Co(NCS)_2_(**L**)]*_n_* family for **L** = **3**–**8** and as well as **9** [20] when crystallization conditions are the same, and the appearance of other nets when different solvent systems are used [20,21] indicates that the role of the solvents is a critical factor in directing the assembly while retaining the 4-connecting Co(NCS)_2_ and bis(3,2′:6′,3″-tpy) nodes. For ligand **4**, a change in crystallization solvents from MeOH and 1,2-dichlorobenzene to MeOH and CHCl_3_ resulted in a material which crystallized in the triclinic space group *P*–1 with cell dimensions *a* = 17.6394(3), *b* = 20.7435(4), *c* = 21.1158(4) Å, α = 79.009(2), *β* = 65.2470(10), *γ* = 64.8080(10)°. Preliminary crystallographic data revealed the assembly of a trinodal self-penetrating network analogous to those observed for [Co(NCS)_2_(**10**)]*_n_***^.^***n*MeOH**^.^**3*n*CHCl_3_ and [Co(NCS)_2_(**11**)]*_n_***^.^**0.8*n*MeOH**^.^**1.8*n*CHCl_3_ [21]. We are currently investigating further the effects of solvent on crystal growth in the reactions of Co(NCS)_2_ with ligands structurally related to **3**–**9**.

## 3. Materials and Methods

### 3.1. General

Compounds **3**–**7** [17] and **8** [22] were prepared as previously reported. Co(NCS)_2_ was purchased from Alfa Aesar (Kandel Germany) and was used as received.

FT-IR spectra were recorded on a PerkinElmer UATR Two instrument.

### 3.2. [Co(NCS)_2_(3)]_n_^.^3.5nC_6_H_4_Cl_2_

A solution of Co(NCS)_2_ (1.8 mg, 10 μmol) in MeOH (5 mL) was layered over a 1,2-dichlorobenzene solution (4 mL) of ligand **3** (6.6 mg, 10 μmol). Pink plate-shaped crystals grew after 26 days, and a single crystal was selected for X-ray diffraction. The remaining crystals were analyzed by PXRD and FT-IR spectroscopy.

### 3.3. [Co(NCS)_2_(4)]_n_^.^5.5nC_6_H_4_Cl_2_

A solution of Co(NCS)_2_ (1.8 mg, 10 μmol) in MeOH (5 mL) was layered over a 1,2-dichlorobenzene solution (4 mL) of ligand **4** (6.9 mg, 10 μmol). Pink block-shaped crystals grew after 34 days, and a single crystal was selected for X-ray diffraction. The remaining crystals were analyzed by PXRD and FT-IR spectroscopy.

### 3.4. [Co(NCS)_2_(5)]_n_^.^4nC_6_H_4_Cl_2_

A solution of Co(NCS)_2_ (1.8 mg, 10 μmol) in MeOH (5 mL) was layered over a 1,2- dichlorobenzene solution (4 mL) of ligand **5** (7.1 mg, 10 μmol). Pink block-shaped crystals grew after 21 days, and one X-ray quality crystal was chosen. The remaining crystals were analyzed by PXRD and FT-IR spectroscopy.

### 3.5. [Co(NCS)_2_(6)]_n_^.^3.8nC_6_H_4_Cl_2_

A solution of Co(NCS)_2_ (1.8 mg, 10 μmol) in MeOH (5 mL) was layered over a 1,2-dichlorobenzene solution (4 mL) of ligand **6** (7.4 mg, 10 μmol). Pink block-shaped crystals grew after 24 days, and one X-ray quality crystal was chosen. The remaining crystals were analyzed by PXRD and FT-IR spectroscopy.

### 3.6. [Co(NCS)_2_(7)]_n_^.^3.1nC_6_H_4_Cl_2_

A solution of Co(NCS)_2_ (1.8 mg, 10 μmol) in MeOH (5 mL) was layered over a 1,2-dichlorobenzene solution (4 mL) of ligand **7** (7.7 mg, 10 μmol). Pink block-shaped crystals grew within two months, and one X-ray quality crystal was chosen. The remaining crystals were analyzed by PXRD and FT-IR spectroscopy.

### 3.7. [Co(NCS)_2_(8)]_n_^.^1.6nC_6_H_4_Cl_2_^.^2nMeOH

A solution of Co(NCS)_2_ (1.8 mg, 10 μmol) in MeOH (5 mL) was layered over a 1,2-dichlorobenzene solution (4 mL) of ligand **8** (8.0 mg, 10 μmol). Pink block-shaped crystals grew within two months, and one X-ray quality crystal was chosen. The remaining crystals were analyzed by PXRD and FT-IR spectroscopy.

### 3.8. Crystallography

Single crystal data were collected on a STOE StadiVari Eulerian 4-circle diffractometer (CuKα radiation) equipped with a Dectris Eiger2 1M detector, or using a STOE StadiVari diffractometer equipped with a Pilatus300K detector and with a Metaljet D2 source (GaKα radiation) with data processing using STOE software (X-Area 1.90, STOE, 2020). Structures were solved using Superflip [31,32] and Olex2 [33]. The model was refined with ShelXL v. 2018/3 [34]. All H atoms were included at geometrically calculated positions and refined using a riding model with U_iso_ = 1.2 of the parent atom. Structure analysis and structural diagrams used CSD Mercury 2022.1.0 [30].

In most structures, the sulfur atom of the [NCS]^−^ unit and the *n*-alkyloxy chains suffered from disorder. Details of the treatment of the disorders and site occupancies are given in the Supplementary Materials in the relevant figure captions. The solvent molecules in all the structures were disordered. In [Co(NCS)_2_(**4**)]*_n_***^.^**5.5*n*C_6_H_4_Cl_2_, [Co(NCS)_2_(**7**)]*_n_***^.^**3.1*n*C_6_H_4_Cl_2_ and [Co(NCS)_2_(**8**)]*_n_***^.^**1.6*n*C_6_H_4_Cl_2_**^.^**2*n*MeOH geometrical restraints for the aromatic ring and restraints for the thermal parameters had to be used to treat the 1,2-Cl_2_C_6_H_4_ molecules. A solvent mask was applied to treat part or all of the solvent region in [Co(NCS)_2_(**3**)]*_n_***^.^**3.5*n*C_6_H_4_Cl_2_, [Co(NCS)_2_(**4**)]*_n_***^.^**5.5*n*C_6_H_4_Cl_2_, [Co(NCS)_2_(**7**)]*_n_***^.^**3.1*n*C_6_H_4_Cl_2_ and [Co(NCS)_2_(**8**)]*_n_***^.^**1.6*n*C_6_H_4_Cl_2_**^.^**2*n*MeOH. In each case, the electron density removed was accounted for in terms of added solvent molecules, and these were added to the formulae and all appropriate numbers.

PXRD data were collected at 295 K in transmission mode using a Stoe Stadi P diffractometer equipped with CuKα1 radiation (Ge(111) monochromator and a DECTRIS MYTHEN 1K detector). Whole-pattern profile matching analysis [35,36,37] of the diffraction patterns was performed using the package FULLPROF SUITE (v. January 2021) [37,38] applying a previously determined instrument resolution function based on a NIST640d standard. The structural models were derived from the single crystal X-ray diffraction data. Refined parameters in Rietveld were scale factor, zero shift, lattice parameters, background points, and peak shapes as a Thompson-Cox-Hastings pseudo-Voigt function. Preferred orientations as a March–Dollase multi-axial phenomenological model were incorporated into the analysis.

Thermogravimetric analysis was carried out under nitrogen on a TGA5500 instrument coupled to a Discovery II MS, Cirrus 3 mass spectrometer. A Barchart scanning method in the mass range 10–125 or 12–160 was used, and the temperature of the TGA instrument was initially stabilized at 30 °C. The samples were heated to 210 °C, and this was maintained for 30 min.

[Co(NCS)_2_(**3**)]*_n_***^.^**3.5*n*C_6_H_4_Cl_2_: C_65_H_50_Cl_7_CoN_8_O_2_S_2_, *M_r_* = 1346.33, pink plate, monoclinic, space group *P*2_1_/*c*, *a* = 14.7191(2) Å, *b* = 15.0949(2) Å, *c* = 16.6847(2) Å, *β* = 115.5830(10)°, *V* = 3343.62(8) Å^3^, *D*_c_ = 1.337 g cm^−3^, *T* = 150 K, *Z* = 2, *µ*(CuK*_α_*) = 5.556 mm^−1^, 52602 reflections measured, 6247 unique (*R*_int_ = 0.0451). Refinement of 5968 reflections (259 parameters) with *I* > 2*σ(I)* converged at final *R*_1_ = 0.1150 (*R*_1_ all data = 0.1178), *wR*_2_ = 0.3133 (*wR*_2_ all data = 0.3159), gof = 1.052. CCDC 2180678.

[Co(NCS)_2_(**4**)]_n_**^.^**5.5*n*C_6_H_4_Cl_2_: C_152.34_H_135.33_Cl_10.67_Co_2_N_16_O_4_S_4_, *M_r_* = 2878.52, pink block, monoclinic, space group *P*2_1_/*c*, *a* = 15.0516(10) Å, *b* = 14.9277(5) Å, *c* = 46.097(2) Å, *β* = 98.736(4)°, *V* = 10237.1(9) Å^3^, *D*_c_ = 1.401 g cm^−3^, *T* = 150 K, *Z* = 3, *µ*(CuK*_α_*) = 4.895 mm^−1^, 80876 reflections measured, 19075 unique (*R*_int_ = 0.0550). Refinement of 12630 reflections (1014 parameters) with *I* > 2*σ(I)* converged at final *R*_1_ = 0.1267 (*R*_1_ all data = 0.1699), *wR*_2_ = 0.3098 (*wR*_2_ all data = 0.3534), gof = 1.082. CCDC 2180679.

[Co(NCS)_2_(**5**)]*_n_***^.^**4*n*C_6_H_4_Cl_2_: C_72_H_60_Cl_8_CoN_8_O_2_S_2_, *M_r_* = 1475.93, pink block, monoclinic, space group *P*2_1_/*n*, *a* = 15.3929(3) Å, *b* = 14.4011(4) Å, *c* = 16.7954(4) Å, *β* = 112.716(2)°, *V* = 3434.31(15) Å^3^, *D*_c_ = 1.427 g cm^−3^, *T* = 150 K, *Z* = 2, *µ*(GaK*_α_*) = 3.926 mm^−1^, 29543 reflections measured, 6585 unique (*R*_int_ = 0.0594). Refinement of 6270 reflections (392 parameters) with *I* > 2*σ(I)* converged at final *R*_1_ = 0.0853 (*R*_1_ all data = 0.0881), *wR*_2_ = 0.2228 (*wR*_2_ all data = 0.2251), gof = 1.048. CCDC 2180677.

[Co(NCS)_2_(**6**)]*_n_***^.^**3.8*n*C_6_H_4_Cl_2_: C_72.80_H_63.20_Cl_7.60_CoN_8_O_2_S_2_, *M_r_* = 1474.58, pink block, monoclinic, space group *P*2_1_/*n*, *a* = 15.4135(4) Å, *b* = 14.3925(2) Å, *c* = 16.9214(4) Å, *β* = 112.398(2)°, *V* = 3470.63(14) Å^3^, *D*_c_ = 1.411 g cm^−3^, *T* = 150 K, *Z* = 2, *µ*(GaK*_α_*) = 3.791 mm^−1^, 24990 reflections measured, 6666 unique (*R*_int_ = 0.0371). Refinement of 6327 reflections (496 parameters) with *I* > 2*σ(I)* converged at final *R*_1_ = 0.0578 (*R*_1_ all data = 0.0603), *wR*_2_ = 0.1523 (*wR*_2_ all data = 0.1551), gof = 1.050. CCDC 2180674.

[Co(NCS)_2_(**7**)]*_n_***^.^**3.1*n*C_6_H_4_Cl_2_: C_70.60_H_64.40_Cl_6.20_CoN_8_O_2_S_2_, *M_r_* = 1399.74, pink block, monoclinic, space group *P*2_1_/*n*, *a* = 15.2267(4) Å, *b* = 14.6023(3) Å, *c* = 16.9263(5) Å, *β* = 114.392(2)°, *V* = 3427.55(16) Å^3^, *D_c_* = 1.356 g cm^−3^, *T* = 150 K, *Z* = 2, *µ*(CuK*_α_*) = 5.159 mm^−1^, 28723 reflections measured, 6654 unique (*R*_int_ = 0.0307). Refinement of 6173 reflections (390 parameters) with *I* > 2*σ(I)* converged at final *R*_1_ = 0.0985 (*R*_1_ all data = 0.1033), *wR*_2_ = 0.2408 (*wR*_2_ all data = 0.2452), gof = 1.024. CCDC 2180676.

[Co(NCS)_2_(**8**)]*_n_***^.^**1.6*n*C_6_H_4_Cl_2_**^.^**2*n*MeOH: C_65.60_H_70.40_Cl_3.20_CoN_8_O_4_S_2_, *M_r_* = 1271.39, pink block, monoclinic, space group *P*2_1_/*n*, *a* = 15.2229(2) Å, *b* = 14.5275(2) Å, *c* = 16.8735(2) Å, *β* = 113.7470(10)°, *V* = 3415.64(8) Å^3^, *D_c_* = 1.236 g cm^−3^, *T* = 150 K, *Z* = 2, *µ*(CuK*_α_*) = 4.090 mm^−1^, 30773 reflections measured, 6692 unique (*R*_int_ = 0.0394). Refinement of 6169 reflections (315 parameters) with *I* > 2*σ(I)* converged at final *R*_1_ = 0.1542 (*R*_1_ all data = 0.1608), *wR*_2_ = 0.3216 (*wR*_2_ all data = 0.3251), gof = 0.999. CCDC 2180675.

## 4. Conclusions

We have reported the single-crystal structures of [Co(NCS)_2_(**3**)]*_n_***^.^**3.5*n*C_6_H_4_Cl_2_, [Co(NCS)_2_(**4**)]_n_**^.^**5.5*n*C_6_H_4_Cl_2_, [Co(NCS)_2_(**5**)]*_n_***^.^**4*n*C_6_H_4_Cl_2_, [Co(NCS)_2_(**6**)]*_n_***^.^**3.8*n*C_6_H_4_Cl_2_, [Co(NCS)_2_(**7**)]*_n_***^.^**3.1*n*C_6_H_4_Cl_2_, and [Co(NCS)_2_(**8**)]*_n_***^.^**1.6*n*C_6_H_4_Cl_2_**^.^**2*n*MeOH, in which the ligands **3**–**8** are bis(3,2′:6′,3″-tpy) ligands with *n*-alkyloxy substituents ranging from *n*-propyl to *n*-octyl. Crystals were grown by layering a MeOH solution of Co(NCS)_2_ over a 1,2-Cl_2_C_6_H_4_ solution of **3**–**8**. For each compound, the assembly of a (6^5^.8) *cds* net was observed. Despite the increasing steric demands of the ligands, the network remains little perturbed, and the *n-*alkyloxy chains (all in extended) are accommodated in cavities in the lattice with a concomitant decrease in the solvent-accessible void space within the net. The assembly of the *cds* net rather than other possible nets is critically dependent upon the choice of solvents for the crystal growth. We are currently exploring the effects of using different solvent systems and will report on these findings in the near future.

## Figures and Tables

**Table 1:** Important bond parameters in the cobalt(II) coordination compounds.

Compound	Co–N_NCS_/Å	Co–N_py_/Å	Range of N–Co–N Bond Angles ^a^/deg
[Co(NCS)_2_(3)]*_n_*^.^3.5*n*C_6_H_4_Cl_2_	2.077(4)	2.151(4), 2.245(4)	87.82(15)–92.18(15)
[Co(NCS)_2_(4)]*_n_*^.^5.5*n*C_6_H_4_Cl_2_	2.069(5), 2.051(5), 2.069(5)	2.149(5), 2.262(5), 2.175(5), 2.288(5), 2.176(5), 2.253(5)	87.60(18)–91.64(18)
[Co(NCS)_2_(5)]*_n_*^.^4*n*C_6_H_4_Cl_2_	2.062(3)	2.249(3), 2.182(3)	89.29(12)–90.71(12)
[Co(NCS)_2_(6)]*_n_*^.^3.8*n*C_6_H_4_Cl_2_	2.0633(19)	2.1854(19), 2.2469(19)	89.12(7)–90.88(7)
[Co(NCS)_2_(7)]*_n_*^.^3.1*n*C_6_H_4_Cl_2_	2.059(3)	2.181(3), 2.255(3)	88.62(12)–91.38(12)
[Co(NCS)_2_(8)]*_n_*^.^1.6*n*C_6_H_4_Cl_2_^.^2*n*MeOH	2.068(5)	2.182(5), 2.243(4)	88.58(18)–91.42(18)

^a^ Only the *cis* angles are given.

**Table 2:** Angles between the least-squares planes of pairs of connected rings in coordinated ligands.

Compound	py-py/°	py_central_-Arene Spacer/°
[Co(NCS)_2_(3)]*_n_*^.^3.5*n*C_6_H_4_Cl_2_	12.9, 28.7	48.4
[Co(NCS)_2_(4)]*_n_*^.^5.5*n*C_6_H_4_Cl_2_	16.0, 24.6; ^a^ 10.6, 24.9; ^b^ 17.5. 22.8 ^c^	46.8 ^a^; 46.8 ^b^; 50.0 ^c^
[Co(NCS)_2_(5)]*_n_*^.^4*n*C_6_H_4_Cl_2_	10.0, 28.1	52.3
[Co(NCS)_2_(6)]*_n_*^.^3.8*n*C_6_H_4_Cl_2_	8.1, 28.3	52.8
[Co(NCS)_2_(7)]*_n_*^.^3.1*n*C_6_H_4_Cl_2_	11.1, 24.7	50.3
[Co(NCS)_2_(8)]*_n_*^.^1.6*n*C_6_H_4_Cl_2_^.^2*n*MeOH	10.8, 25.7	51.4

^a^ For the tpy unit containing N1N2N3; ^b^ tpy unit with N4N5N6; ^c^ tpy unit with N7N8N9.

**Table 3:** The calculated (see text) solvent-accessible void space in solvent-free [Co(NCS)_2_(**L**)]*_n_* structures.

**Ligand, L**	**3**	**4**	**5**	**6**	**7**	**8**
**Void space/%**	49.3	44.5	43.1	39.8	35.6	34.4

**Table 4:** Results of thermogravimetric analyses.

Compound	Initial Weight /mg	Weight Loss /mg	Weight Loss /%	Calculated Solvent Molecules ^a^
[Co(NCS)_2_(**3**)]*_n_***^.^**3.5*n*C_6_H_4_Cl_2_	1.14	0.45	39.5	3.7 mol C_6_H_4_Cl_2_
[Co(NCS)_2_(**4**)]*_n_***^.^**5.5*n*C_6_H_4_Cl_2_	0.76	0.30	39.5	3.8 mol C_6_H_4_Cl_2_
[Co(NCS)_2_(**6**)]*_n_***^.^**3.8*n*C_6_H_4_Cl_2_	1.74	0.67	38.5	3.9 mol C_6_H_4_Cl_2_

^a^ Per formula unit.

## Data Availability

Original data will be placed on zenodo.org after paper publication.

## Supplementary Materials

The following supporting information can be downloaded at: https://www.mdpi.com/article/10.3390/molecules27154995/s1, Figures S1–S6: Structural figures with atom numbering; Figures S7–S12: solid-state IR spectra of the coordination compounds. Figure S13: Overlay of the experimental (blue) PXRD (298 K) for the bulk material and that predicted (black) from the single crystal structure (150 K) of [Co(NCS)_2_(**3**)]_n_**^.^**3.5nC_6_H_4_Cl_2_. Figure S14: Overlay of the experimental (blue) PXRD (298 K) for the bulk material and that predicted (black) from the single crystal structure (150 K) of [Co(NCS)_2_(**4**)]_n_**^.^**5.5nC_6_H_4_Cl_2_. Figure S15: TGA and mass spectrometric traces for the analysis of [Co(NCS)_2_(**3**)]_n_**^.^**3.5nC_6_H_4_Cl_2_. Red: temperature vs. time; black: weight of sample vs. time; dark blue: mass detection for *m*/*z* 146, 148 and 111; orange: mass detection for *m*/*z* 31.0. Figure S16: TGA and mass spectrometric traces for the analysis of [Co(NCS)_2_(**4**)]_n_**^.^**5.5nC_6_H_4_Cl_2_. Red: temperature vs. time; black: weight of sample vs. time; dark blue: mass detection for *m*/*z* 146, 148 and 111; orange: mass detection for *m*/*z* 31.0. Figure S17: TGA and mass spectrometric traces for the analysis of [Co(NCS)_2_(**6**)]_n_**^.^**3.8nC_6_H_4_Cl_2_. Red: temperature vs. time; black: weight of sample vs. time; dark blue: mass detection for *m*/*z* 146, 148 and 111; orange: mass detection for *m*/*z* 31.0.

## Sample Availability

No samples are available.
