# Supplementary material for: A Tail Does Not Always Make a Difference: Assembly of cds Nets from Co(NCS)2 and 1,4-bis(n-Alkyloxy)-2,5-bis(3,2′:6′,3″-terpyridin-4′-yl)benzene Ligands
Source: Molecules. 2022 Aug 5;27(15):4995. doi: 10.3390/molecules27154995 (PMC9370426; doi:10.3390/molecules27154995)
Supplement: Supplementary file 1 [file molecules-27-04995-s001.zip › Supplementary Materials.pdf]

Supplementary Materials to accompany

A Tail Does Not Always Make a Difference: Assembly of *cds* Nets from Co(NCS)$_2$ and 1,4-bis(*n*-Alkyloxy)-2,5-bis(3,2′:6′,3′′-terpyridin-4′-yl)benzene Ligands

Simona S. Capomolla, Giacomo Manfroni, Alessandro Prescimone, Edwin C. Constable and Catherine E. Housecroft *

Department of Chemistry, University of Basel, BPR 1096, Mattenstrasse 24a,
CH-4058 Basel, Switzerland

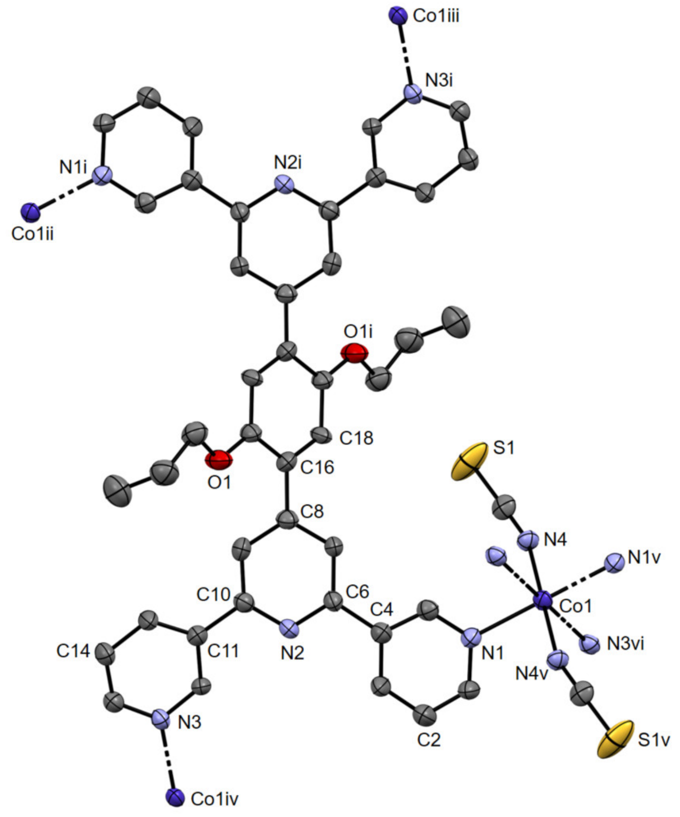

Figure S1. The structure of the asymmetric unit in $[Co(NCS)_2(\mathbf{3})]_n \cdot 3.5nC_6H_4Cl_2$, with symmetry generated atoms. Hydrogen atoms are omitted for clarity; ellipsoids are plotted at 50% probability level. Symmetry codes: i = –1–*x*, –1–*y*, –1–*z*; ii = 1+*x*, *y*, 1+*z*; iii= –1–*x*, $^1/_2$+*y*, –$^3/_2$–*z*; iv = –2–*x*, –$^1/_2$+*y*, –$^3/_2$–*z*; v = –2–*x*, –1–*y*, –2–*z*; vi= –2–*x*, $^1/_2$+*y*, –$^3/_2$–*z*.

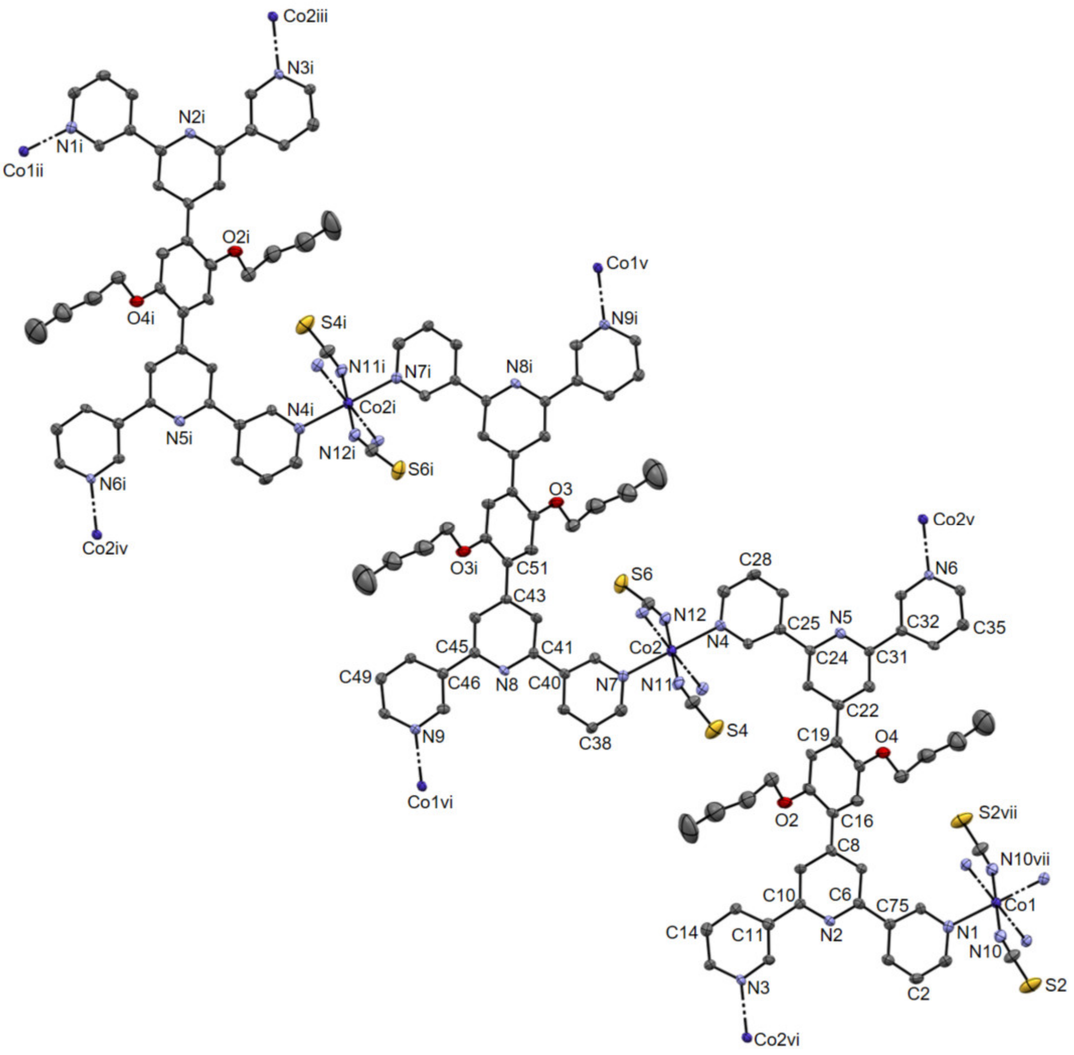

Figure S2. The structure of the asymmetric unit in $[Co(NCS)_2(\mathbf{4})]_n{\cdot}5.5nC_6H_4Cl_2$, with symmetry generated atoms. Hydrogen atoms and solvent molecules are omitted for clarity; ellipsoids are plotted at 50% probability level. Symmetry codes: i = 1–*x*, 1–*y*, 2–*z*; ii = –1+*x*, *y*, 1+*z*; iii = –1+*x*, $^1/_2$–*y*, $^1/_2$+*z*; iv = *x*, $^1/_2$–*y*, $^1/_2$+*z*; v = 1–*x*, –$^1/_2$+*y*, $^3/_2$–*z*; vi = 2–*x*, $^1/_2$+*y*, $^3/_2$–*z*; vii = 2–*x*, 1–*y*, 1–*z*. The sulfur atoms S2, S4 and S6 of the $[NCS]^-$ units are disordered and have been modelled over sites of 50:30:20%, 60:20:20% and 70:30% occupancies, respectively, and only the major occupancy site is shown. The terminal unit in the –OButyl chain on O3 is disordered and has been modelled over sites of 75% and 25% occupancies and only the major occupancy site is shown.

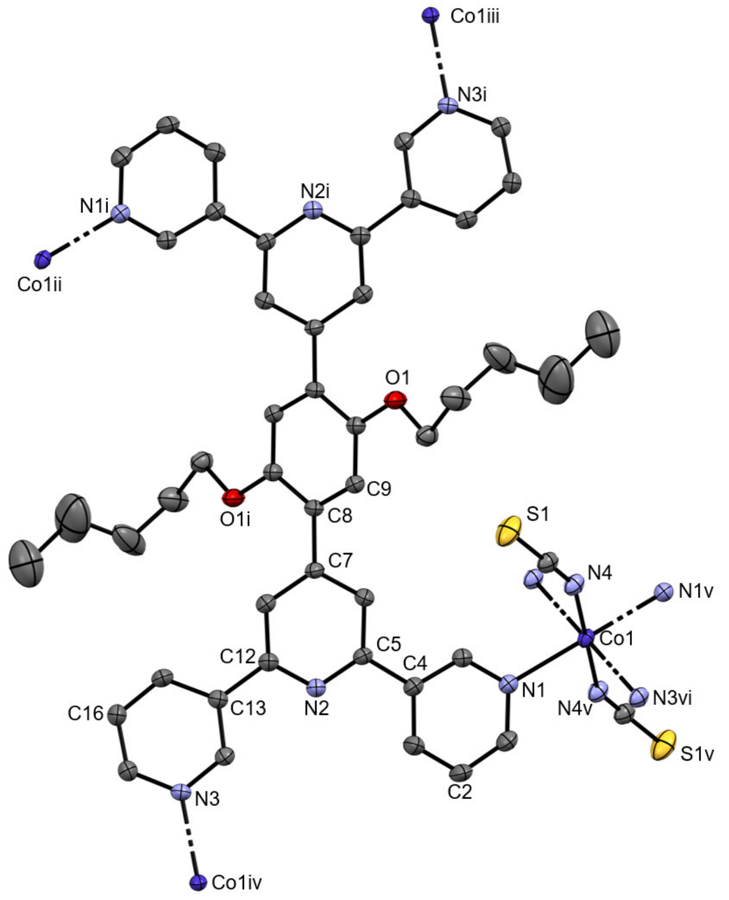

Figure S3. The structure of the asymmetric unit in $[Co(NCS)_2(\mathbf{5})]_n \cdot 4nC_6H_4Cl_2$, with symmetry generated atoms. Hydrogen atoms and solvent molecules are omitted for clarity; ellipsoids are plotted at 50% probability level. Symmetry codes: i = 1–*x*, 1–*y*, 1–*z*; ii = *x*, *y*, 1+*z*; iii = 1/2–*x*, 1/2+*y*, 1/2–*z*; iv = 3/2–*x*, –1/2 + *y*, 1/2–*z*; v = 1–*x*, 1–*y*, –*z*; vi = 3/2–*x*, ½+*y*, 1/2–*z*.

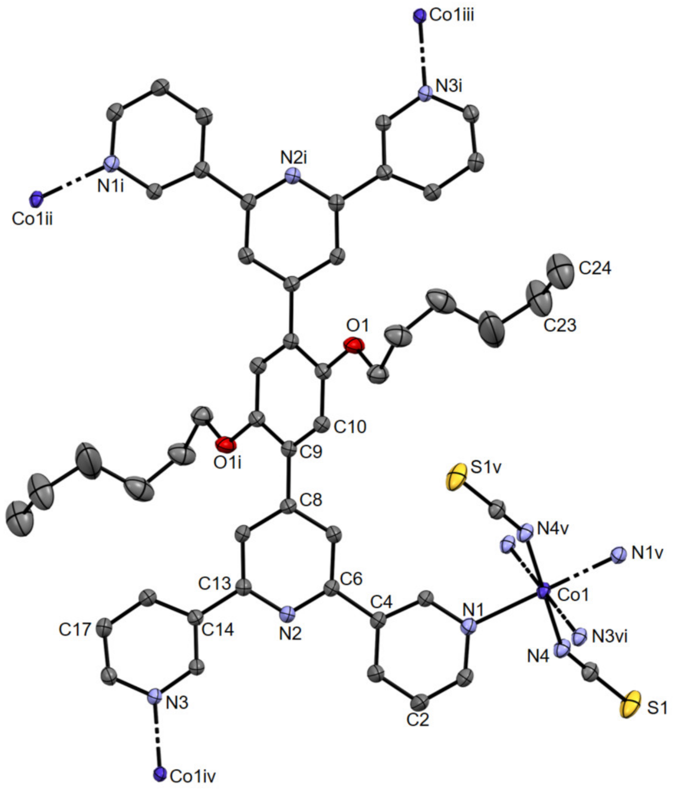

Figure S4. The structure of the asymmetric unit in $[Co(NCS)_2(\mathbf{6})]_n \cdot 3.8nC_6H_4Cl_2$, with symmetry generated atoms. Hydrogen atoms and solvent molecules are omitted for clarity; ellipsoids are plotted at 50% probability level. Symmetry codes: i = 1–*x*, 1–*y*, 2–*z*; ii = *x*, *y*, 1+*z*; iii = $^1/_2$–*x*, ½+*y*, $^3/_2$–*z*; iv = $^3/_2$–*x*, –$^1/_2$+*y*, $^3/_2$–*z*; v = 1–*x*, 1–*y*, 1–*z*; vi = $^3/_2$–*x*, $^1/_2$+*y*, $^3/_2$–*z*. The terminal units with C23 and C24 in the –OHexyl chain are disordered and have been modelled over sites of 60% and 40% occupancies and only the major one is shown.

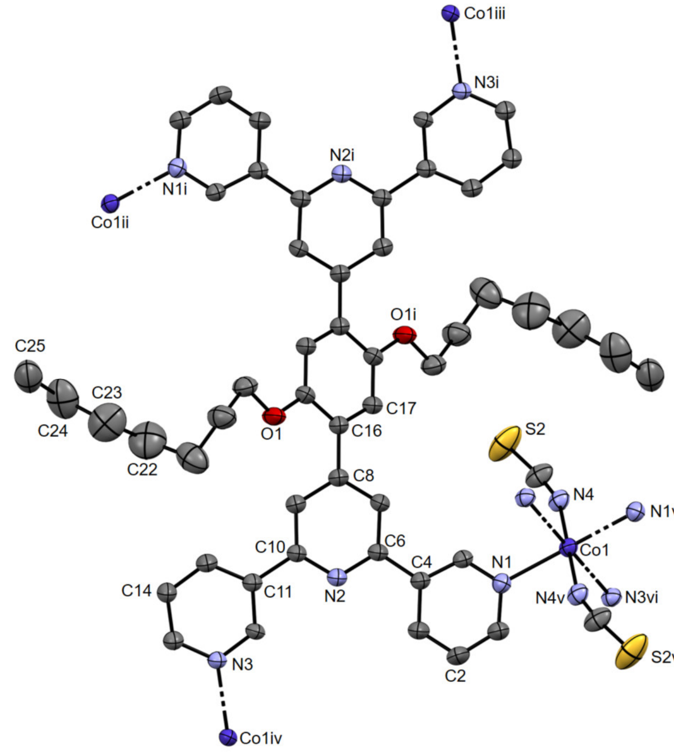

Figure S5. The structure of the asymmetric unit in $[Co(NCS)_2(\mathbf{7})]_n \cdot 3.1nC_6H_4Cl_2$, with symmetry generated atoms. Hydrogen atoms and solvent molecules are omitted for clarity; ellipsoids are plotted at 50% probability level. Symmetry codes: i = 1–*x*, 1–*y*, 1–*z*; ii = *x*, *y*, –1+*z*; iii = $^3/_2$–*x*, –$^1/_2$+*y*, $^3/_2$–*z*; iv = $^1/_2$–*x*, $^1/_2$+*y*, $^3/_2$–*z*; v = 1–*x*, 1–*y*, 2–*z*; vi = $^1/_2$–*x*, –$^1/_2$+*y*, $^3/_2$–*z*. The terminal units with C22, C23, C24 and C25 in the –OHeptyl chain are disordered and have been modelled over sites of 50% and 50% occupancies and only one of the equal occupancy sites is shown. The sulfur S2 of the $[NCS]^-$ unit is disordered and has been modelled over sites of 80% and 20% occupancies and only the major occupancy site is shown.

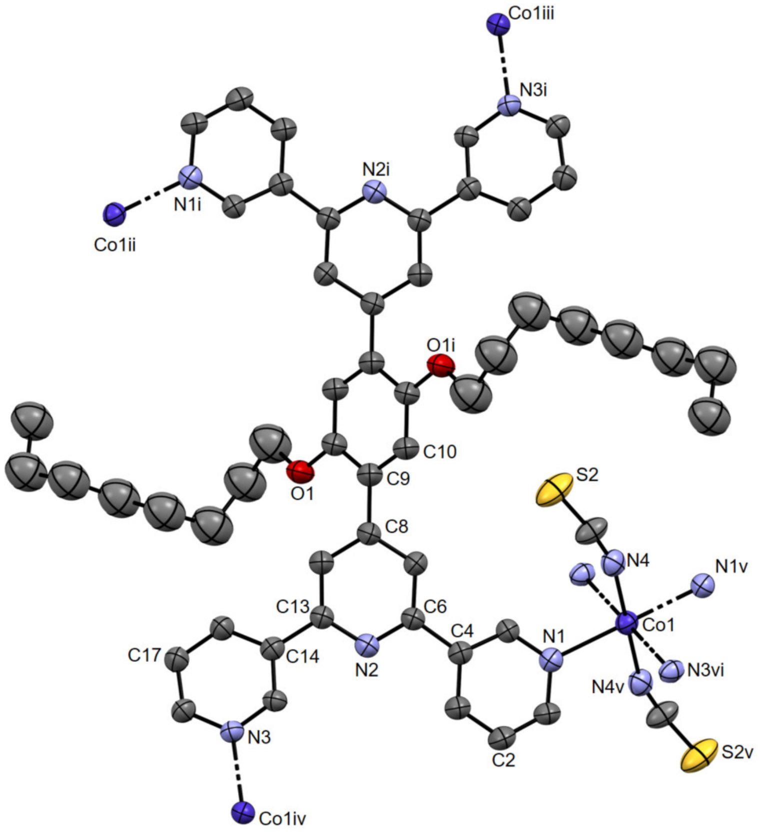

Figure S6. The structure of the asymmetric unit in $[Co(NCS)_2(\mathbf{8})]_n{\cdot}1.6nC_6H_4Cl_2{\cdot}2n$MeOH, with symmetry generated atoms. Hydrogen atoms and solvent molecules are omitted for clarity; ellipsoids are plotted at 50% probability level. Symmetry codes: i = –x, 1–y, 1–z; ii = x, y, 1+z; iii = –$^1/_2$–x, $^1/_2$+y, $^1/_2$–z; iv = $^1/_2$–x, –$^1/_2$+y, $^1/_2$–z; v = –x, 1–y, –z; vi = $^1/_2$–x, $^1/_2$+y, $^1/_2$–z. The sulfur atom S2 of the $[NCS]^-$ unit is disordered and has been modelled over sites of 80% and 20% occupancies and only the major occupancy site is shown.

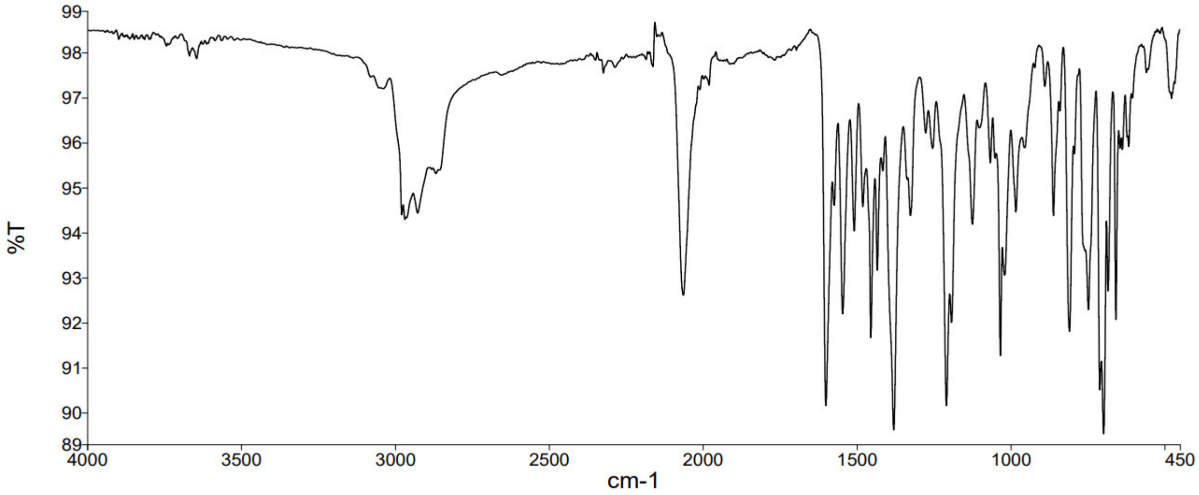

Figure S7. The solid-state FT-IR spectrum of $[Co(NCS)_2(\mathbf{3})]_n{\cdot}3.5nC_6H_4Cl_2$.

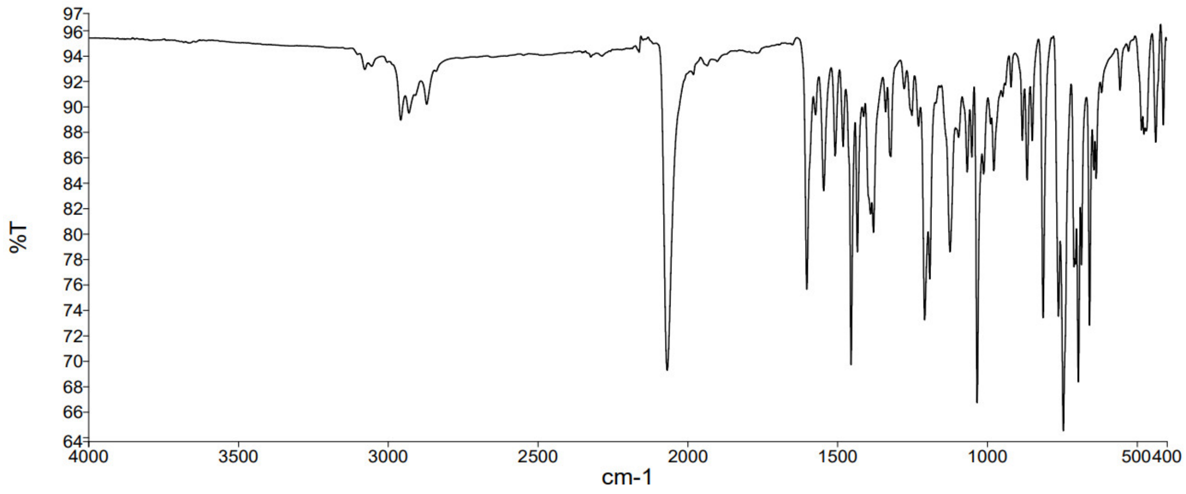

Figure S8. The solid-state FT-IR spectrum of $[Co(NCS)_2(\mathbf{4})]_n{\cdot}5.5nC_6H_4Cl_2$.

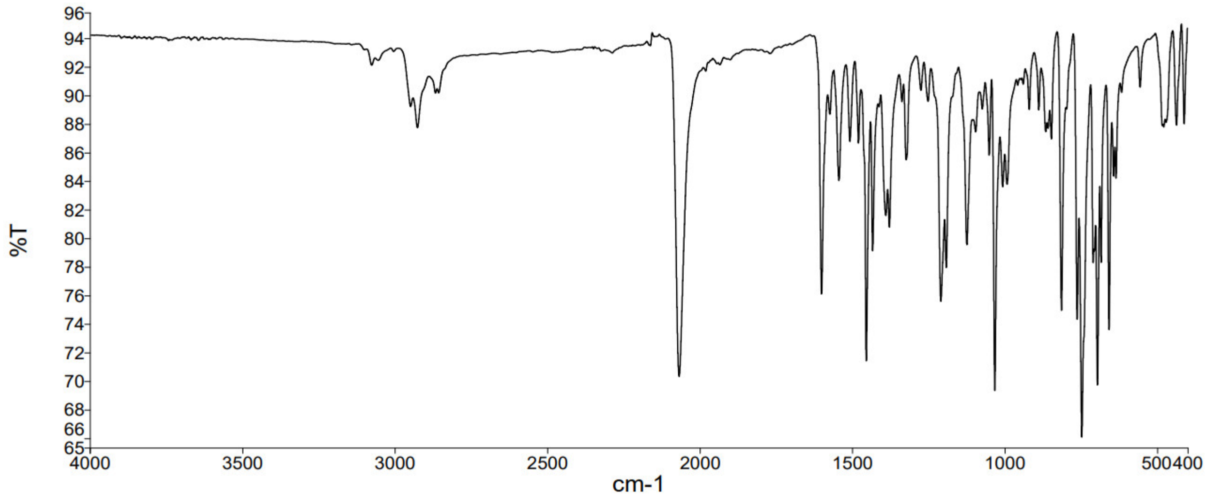

Figure S9. The solid-state FT-IR spectrum of $[Co(NCS)_2(\mathbf{5})]_n{\cdot}4nC_6H_4Cl_2$.

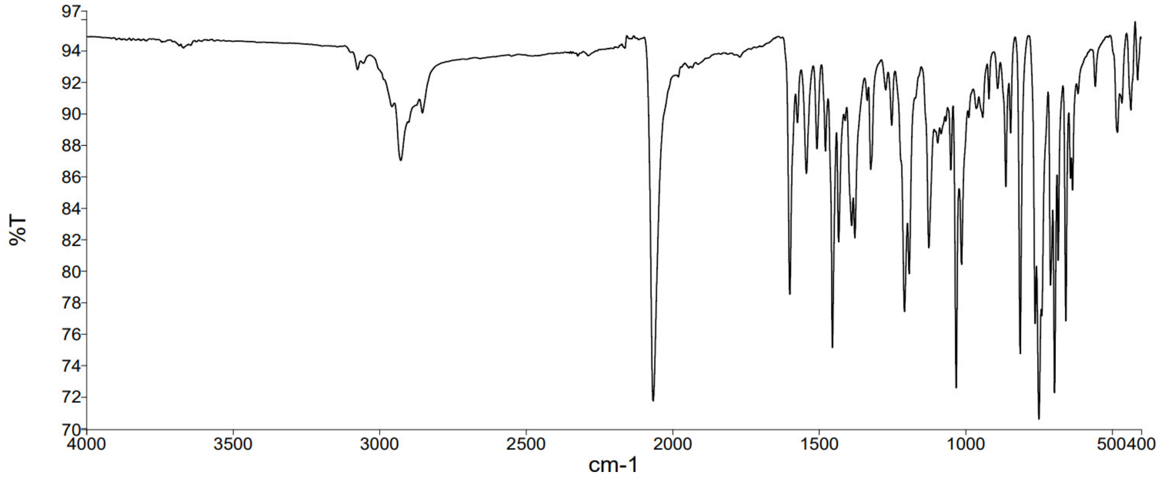

Figure S10. The solid-state FT-IR spectrum of $[Co(NCS)_2(\mathbf{6})]_n{\cdot}3.8nC_6H_4Cl_2$.

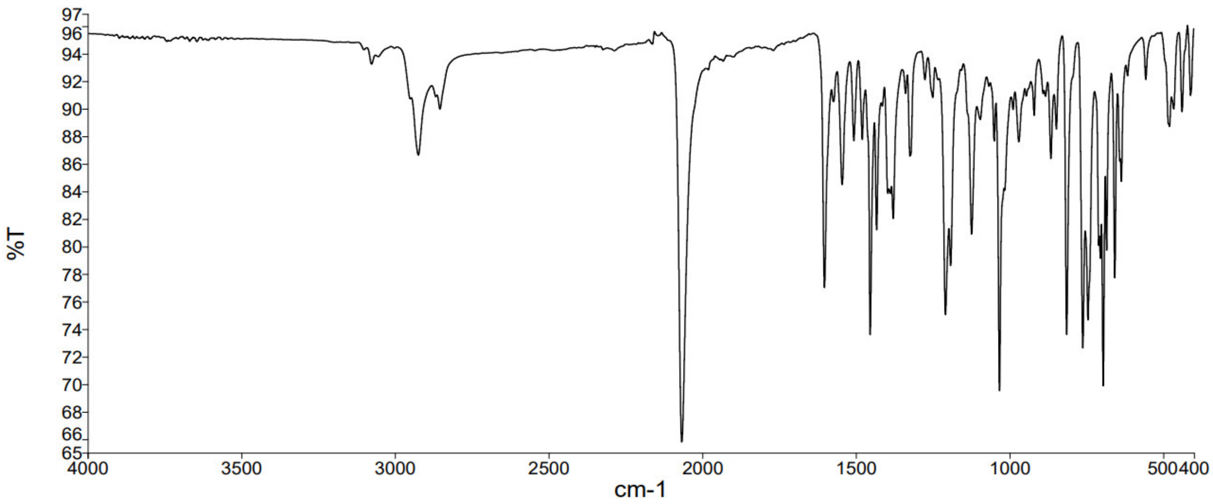

Figure S11. The solid-state FT-IR spectrum of $[Co(NCS)_2(\mathbf{7})]_n{\cdot}3.1nC_6H_4Cl_2$.

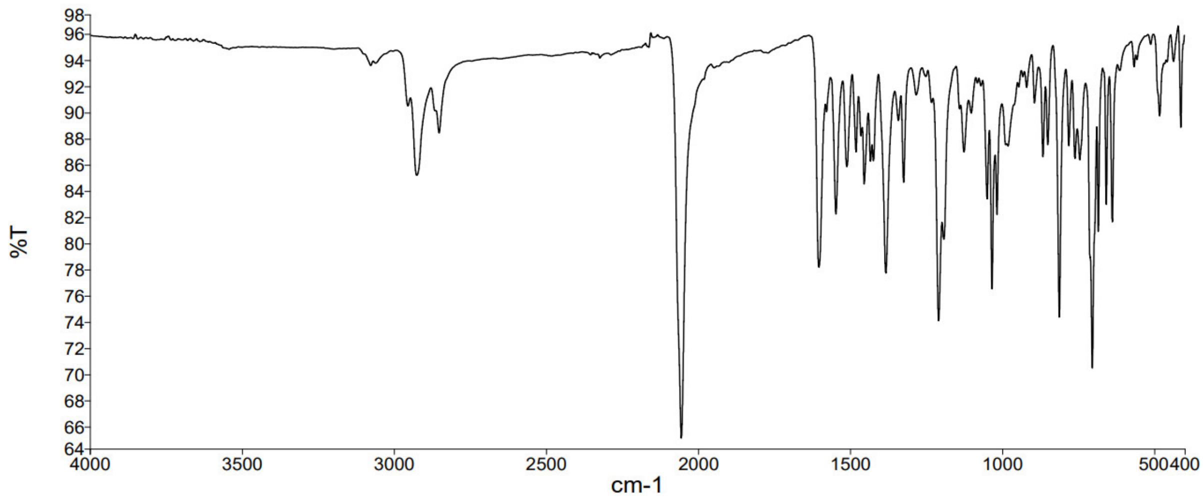

Figure S12. The solid-state FT-IR spectrum of $[Co(NCS)_2(\mathbf{8})]_n{\cdot}1.6nC_6H_4Cl_2{\cdot}2nMeOH$.

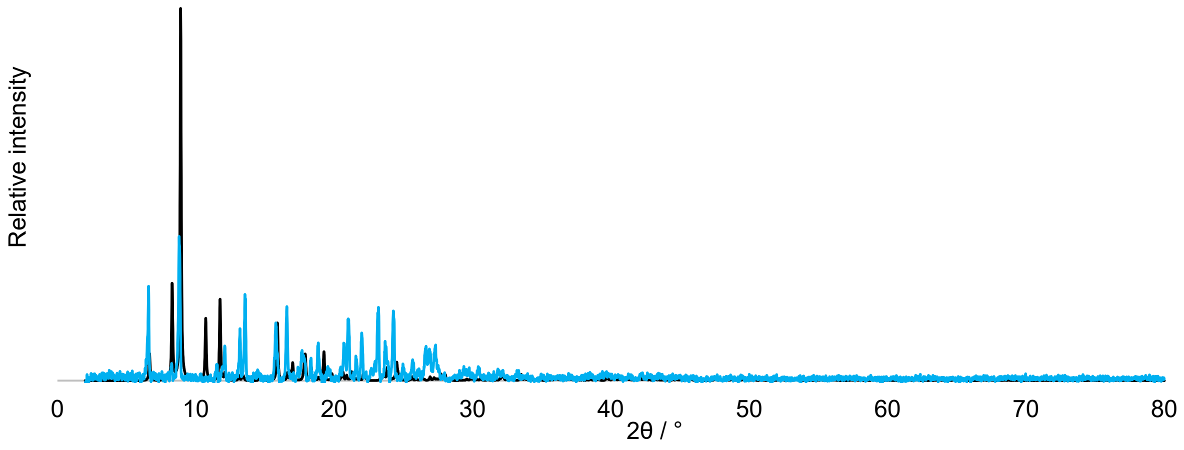

Figure S13. Overlay of the experimental (blue) PXRD (298 K) for the bulk material and that predicted (black) from the single crystal structure (150 K) of $[Co(NCS)_2(\mathbf{3})]_n{\cdot}3.5nC_6H_4Cl_2$.

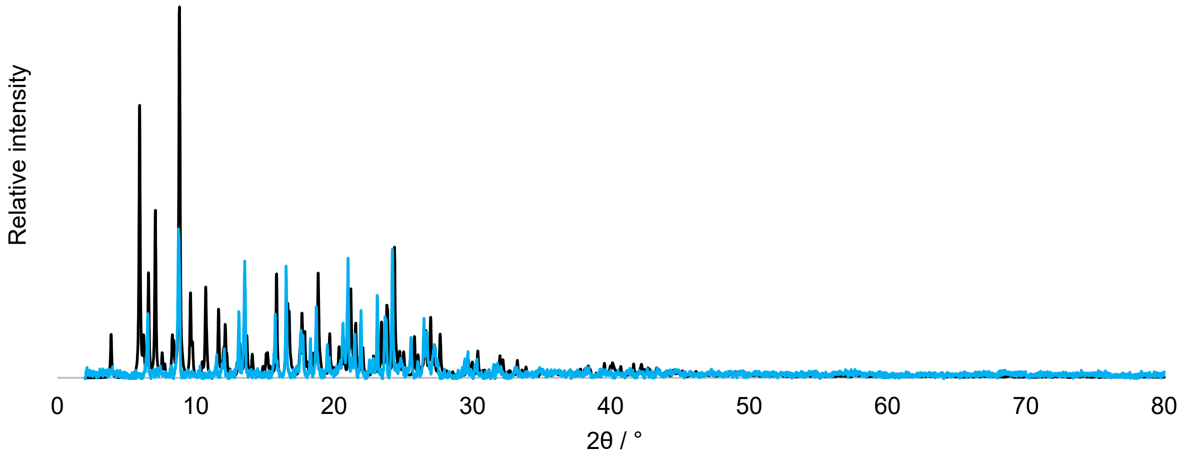

Figure S14. Overlay of the experimental (blue) PXRD (298 K) for the bulk material and that predicted (black) from the single crystal structure (150 K) of $[Co(NCS)_2(\mathbf{4})]_n \cdot 5.5nC_6H_4Cl_2$.

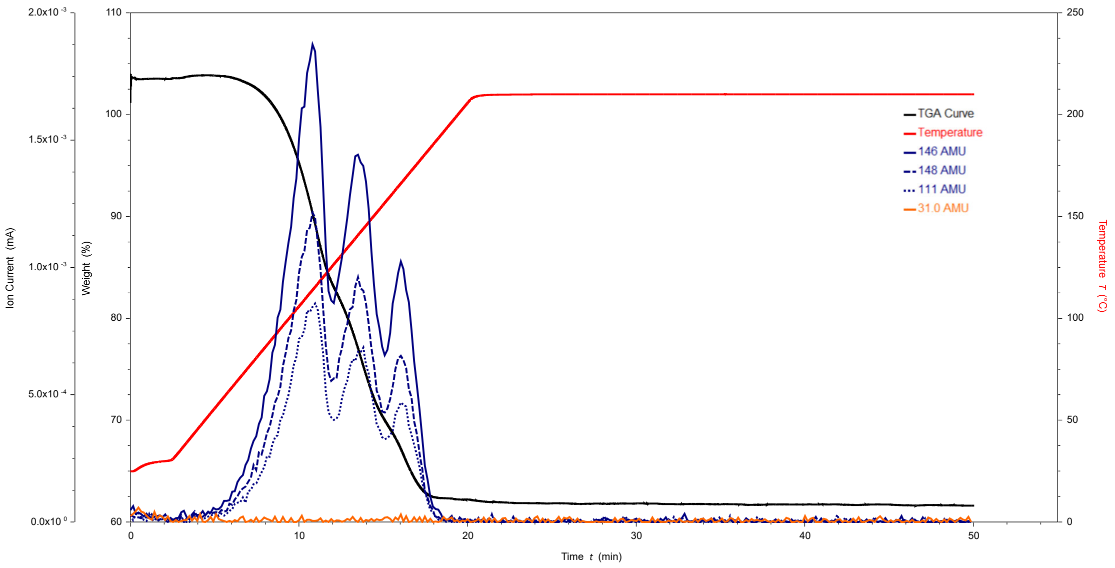

Figure S15. TGA and mass spectrometric traces for the analysis of $[Co(NCS)_2(\mathbf{3})]_n \cdot 3.5nC_6H_4Cl_2$. Red: temperature vs. time; black: weight of sample vs. time; dark blue: mass detection for *m*/*z* 146, 148 and 111; orange: mass detection for *m*/*z* 31.0.

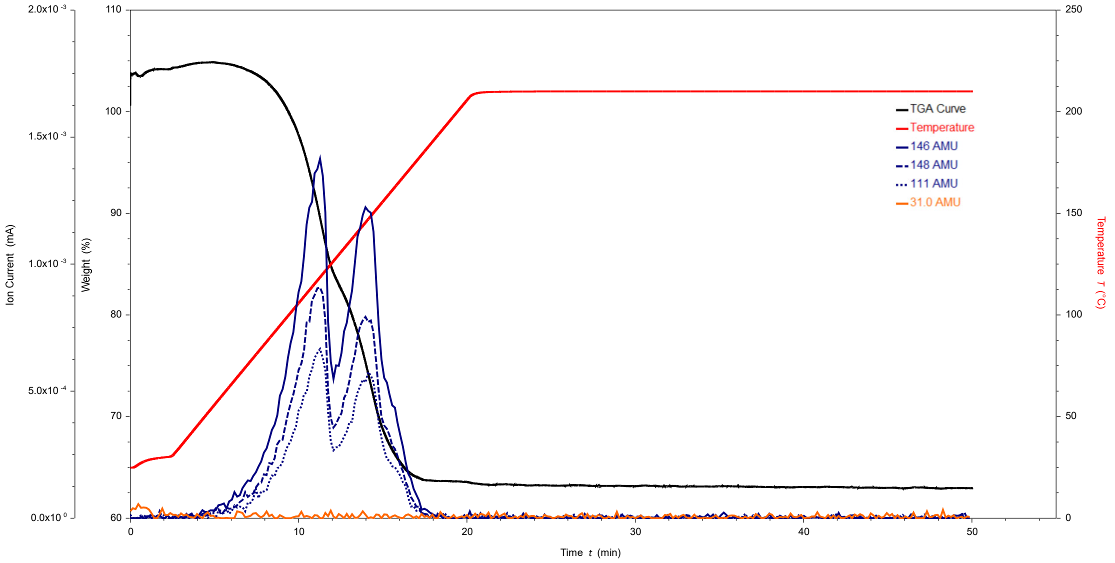

Figure S16.TGA and mass spectrometric traces for the analysis of $[Co(NCS)_2(\mathbf{4})]_n{\cdot}5.5nC_6H_4Cl_2$. Red: temperature vs. time; black: weight of sample vs. time; dark blue: mass detection for *m/z* 146, 148 and 111; orange: mass detection for *m/z* 31.0.

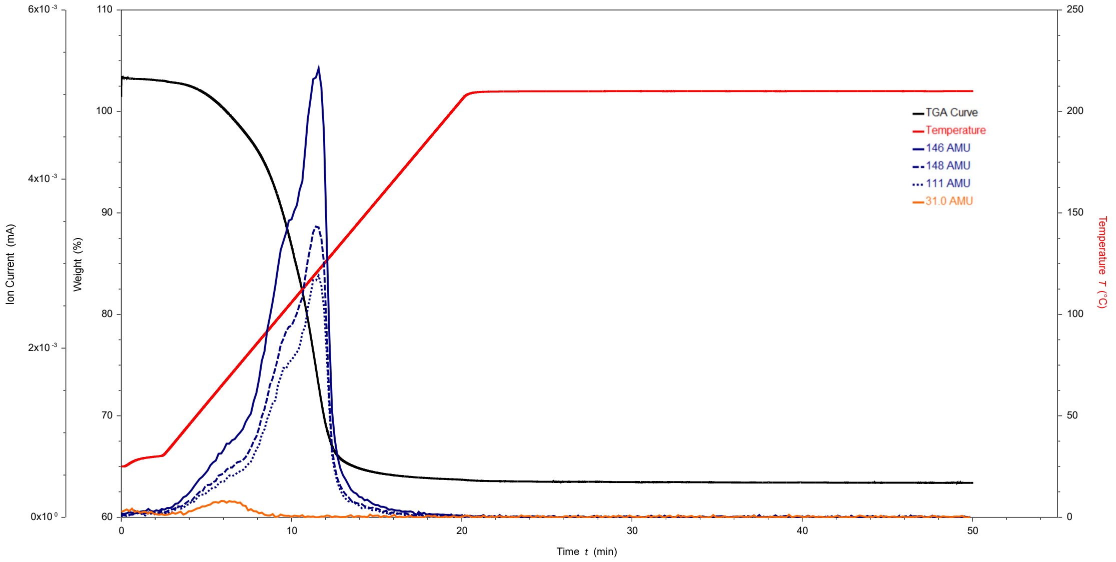

Figure S17. TGA and mass spectrometric traces for the analysis of $[Co(NCS)_2(\mathbf{6})]_n{\cdot}3.8nC_6H_4Cl_2$. Red: temperature vs. time; black: weight of sample vs. time; dark blue: mass detection for *m/z* 146, 148 and 111; orange: mass detection for *m/z* 31.0.
